# Supplementary material for: How does China’s green factory policy affect substantive green innovation?
Source: PLoS One. 2025 Jun 4;20(6):e0325438. doi: 10.1371/journal.pone.0325438 (PMC12136330; doi:10.1371/journal.pone.0325438)
Supplement: Attachment — Submitted filename: Review Comments.pdf [file pone.0325438.s001.pdf]

The manuscript (PONE-D-24-52239) entitled “How does China’s green factory policy affect substantive green innovation?” provides an empirical analysis on studying the nexus between China's Green factory policy and green innovation using the firm-level panel data collected between 2012 and 2021. The authors mainly utilized staggered difference-in-differences to establish the causality. The main results show that green factory evaluation can significantly improve corporate substantive green innovation. Overall, the manuscript is original and interesting. I have several minor suggestions for the authors:

1. The research gap is not identified in a compelling way in the introduction section. *IMPORTANCE, NOVELTY* should be clearly stated and presented in the introduction section. Also, the authors can enrich the contributions to the existing research in the introduction section.

2. Comparisons with other studies have to be provided in the results discussion section. Please interpret and describe the significance of your findings in light of what was already known about the research problem being investigated and explain any new understanding or fresh insights about the problem after you've taken the findings into consideration. Please provide a comparison with other studies.

3. Model construction section of your paper should clearly articulate the reasons why you chose A PARTICULAR PROCEDURE OR TECHNIQUE. The methodology should discuss the problems that were anticipated and the steps you took to prevent them from occurring, such as the heterogeneous treatment effects in running the staggered difference-in-differences regression.

4. Although the authors presented the policy background and main variables empirically used in sections 4 and 5, it appears too sample and unintuitive. I would recommend the authors to detail the variables and results using more beautiful figures, which can make the work appealing for readers in standard international journals.

5. Its topic appears to be within the scope of macro-level pilot policy and corporate green innovation, the authors should cite the following recent research from PLOS ONE or closed journals to connect to this ongoing discourse.

[1] https://doi.org/10.1016/j.eneco.2024.108047

[2] https://doi.org/10.1016/j.jbusres.2023.114147

[3] https://doi.org/10.1016/j.chieco.2024.102236

[4] https://doi.org/10.1016/j.techfore.2022.122007

6. The last section needs to be re-organized in sub-sections structure, with more insightful implications of your findings for managers and researchers (ideally in two separate sections, i.e., theoretical and practical implications). Also, the authors should clearly explain the shortcomings of this article and explore future research directions that can further expand on deification.
