# Supplementary material for: How does China’s green factory policy affect substantive green innovation?
Source: PLoS One. 2025 Jun 4;20(6):e0325438. doi: 10.1371/journal.pone.0325438 (PMC12136330; doi:10.1371/journal.pone.0325438)
Supplement: Attachment — Submitted filename: Response to Reviewers.docx [file pone.0325438.s003.docx]

Dear Reviewer 1,

Thank you very much for giving us the opportunity to further improve the quality of our manuscript entitled **“How does China’s green factory policy affect substantive green innovation?” (PONE-D-24-52239)**. We deeply appreciate your valuable concerns and suggestions, which enable us to further improve our paper. We have read through all comments from you and tried our best to revise the entire paper as suggested. Revised portions are marked with **red words** in the paper. The main corrections in the paper and the responses to your comments are listed as follows:

1) There are widespread concerns about biases in staggered difference-in-differences regression estimators. As mentioned in the paper, “from 2017 to 2020, the MIIT identified five batches of green factory lists and evaluated them at different times, as stated in the General Office of the MIIT’s Notice on Developing Green Manufacturing System and related documents.” The paper covers the period from 2012 to 2021 and employs a staggered difference-in-differences strategy. However, when treatment effects change over time, the standard staggered DD regressions can introduce a “bad comparisons” problem, leading to significant estimates with the wrong sign. The econometric literature has proposed several alternative DD estimation techniques, such as Callaway and Sant'Anna (2021) and Sun and Abraham (2021), to circumvent the problems with TWFE staggered DD estimators. And there are applied studies using these estimators as a remedy. Therefore, I suggest at least one of these estimators be used in this paper (at least in the section of robustness checks) to provide more valid estimates.

- Thank you for your insightful review on our manuscript. The issue you pointed out will help enhance the rigor and standardization of the conclusions from our empirical analysis.
- As suggested by you, we have conducted relevant tests to provide more efficient estimation results, and these modifications are detailed in Section 5.3.2 Heterogeneous treatment effects. Specifically, the revisions are shown below (indicated by red font in the revised manuscript):

**5.3.2 Heterogeneous treatment effects**

Given that GFE policy announcements are not clustered in the same year, varying treatment points over time will subject the traditional two-way fixed effects (TWFE) to potential estimation bias (Sun and Abraham, 2021; Callaway and Sant’Anna, 2021; Goodman-Bacon, 2021). If there is heterogeneity in treatment effects, the TWFE estimator may struggle to accurately identify the true average treatment effect. For example, in our study, the total DID estimator over the sample period can be divided into the following three components: (i) New entrants to the GFE (Treatment) and those who have never been selected for the GFE (Never Treated); (ii) New entrants to the GFE (Earlier Group Treatment) and those who have not yet joined the GFE (Later Group Comparison); (iii) New entrants to the GFE (Later Group Treatment) and those who are already participating in the GFE (Earlier Group Comparison). The total DID estimator is derived as a weighted average of the basic 2$\times$2 DID estimates from the three components mentioned above. Among the three control groups, the first two serve as ‘good controls’ because they assess the impact of whether or not entering the GFE affects firms’ SUGI. However, the treatment effect in the third control is not necessarily homogeneous. If the weights assigned to the control mean estimator of the third are larger, this can influence the results of the two-way fixed effects estimator, thereby introducing bias (Goodman-Bacon, 2021; Sun and Abraham, 2021). To mitigate the bias problem in TDID estimation caused by heterogeneous treatment effects, we employ the following two methods to analyze heterogeneous treatment effects. To address the bias problem in TDID estimation caused by heterogeneous treatment effects, we employ the following two methods to analyze heterogeneous treatment effects and mitigate the limitations of the TWFE estimator.

First, considering the bias diagnosis of the estimator and Goodman-Bacon decomposition. Following Goodman-Bacon (2021), we conducted a bias diagnosis of the TWFE estimator to examine whether the third control group is overrepresented. Fig 6 presents the results of the Goodman-Bacon decomposition, indicating that the third control group constitutes a small proportion in our study. It suggests that the effect of GFE on SUGI is not driven by the third control group. In addition, we provide detailed results of the Goodman-Bacon decomposition in Table 4. It can be observed that the primary source of the overall DID estimates comes from the estimate using the never-treated sample as the control group, which accounts for up to 95% of the weight (Treatment vs. Never Treated). In contrast, the weight of the sample using those who received treatment earlier as the control group is only 0.9% (Later Treatment vs. Earlier Comparison), indicating a minimal impact on the overall estimates. These results suggest that the average treatment effects derived from the benchmark regression results in this study are not significantly biased.

Second, considering the heterogeneity-robust estimator. When the heterogeneous treatment effects are more pronounced, the dynamic effects obtained through the classical event study method may also suffer from estimation bias. To address this issue, some econometricians have employed heterogeneity-robust DID estimators to re-estimate the dynamic effects. This approach has been widely adopted in the existing literature (Braghieri et al., 2022; Liu et al., 2022; Biasi and Sarsons, 2022). Compared to the estimators proposed by De Chaisemartin and D’Haultfoeuille (2020), Callaway and Sant’Anna (2021), and others, this estimator exhibits relatively strong estimation properties and is capable of satisfying unbiasedness, validity, and consistency simultaneously. Based on this, we employ the estimation method proposed by Sun and Abraham (2021) to conduct the dynamic effects test. Fig 7 presents the estimation results, demonstrating that the parallel trend assumption remains valid after addressing the bias problem in TDID estimation caused by heterogeneous treatment effects. This confirms that the benchmark regression results in this study are robust and reliable.

**Fig 6. SUGI.**

**Table 4. Goodman-Bacon decomposition results.**

|  | **(1)** | **(2)** |
| --- | --- | --- |
|  | **SUGI** | |
| **Control Group Type** | **Weights** | **Estimated Value** |
| **Treatment vs. Never treated** | 0.954 | 0.044 |
| **Earlier Treatment vs. Later Comparison** | 0.011 | -0.084 |
| **Later Treatment vs. Earlier Comparison** | 0.009 | -0.013 |

**Fig 7. Parallel trends based on heterogeneity robust DID estimator (Sun and Abraham, 2021).**

- Thank you very much!

2) The criteria for defining green invention patents and their representativeness are questionable. First, the authors do not provide a clear explanation of the standards used to define green invention patents. Given that different classification systems or evaluation frameworks could lead to inconsistencies in the number of green patents filed by firms, this lack of clarity raises concerns about the validity of the measure. Second, it is important to note that not all green invention patents necessarily represent substantial technological innovation. Some patents may involve only minor technical improvements, which may not have significant practical market value. While invention patents are generally considered to be of higher quality, variations in patent quality across firms could lead to biased or inaccurate estimates. Moreover, firms' green innovation activities may include other forms of innovation, such as the adoption of green technologies or improvements in production processes, which are not adequately captured by patent filings alone. I recommend that the authors clarify the criteria used to define green invention patents and consider using more comprehensive and representative indicators to measure firms' actual green innovation.

- Thank you for your insightful review on our manuscript. The issue you pointed out is highly important for improving the quality of our manuscript. SUGI is the core variable of this study, but our explanation for choosing green invention patents to measure SUGI was initially insufficient. Your revisions highlighted this issue, and we have made every effort to refine it. We sincerely thank you for your thorough review and valuable suggestions, which have been immensely helpful to us.
- As suggested by you, we have revised and rewritten Section 4.1.2 Substantive green innovation (SUGI). incorporating the following three main components. First, we have added relevant content to explain in detail the criteria for defining green invention patents. Second, we have explained the rationale for choosing green patents to measure SUGI to enhance the reliability of this indicator. Third, considering other forms of green innovation, we also selected additional indicators to measure SUGI and included them in the robustness tests. Through these approaches, we aim to provide a more comprehensive measure of corporate SUGI and offer reliable empirical evidence supporting the conclusion that GFE promotes SUGI. Specifically, the revisions are shown below (indicated by red font in the revised manuscript):

**4.1.2 Substantive green innovation (SUGI)**

Green patents are an important indicator of the output of green innovation knowledge outcomes for enterprises and are widely used to measure corporate green innovation (Liu et al., 2025; Gao et al., 2025; Tawiah et al., 2024; Huang et al., 2022). They primarily include two types: green invention patents and green utility model patents. Although both types of green patents can be used to measure corporate green innovation, they differ significantly in terms of technical content and the difficulty of filing.

According to the China’s Patent Law, invention patents typically refer to a new technical solution related to a product, a method, or an improvement thereof, whereas utility model patents refer to new technical solutions pertaining to the shape, structure, or combination of products that are suitable for practical application. In terms of examination content, the examination process for invention patents is more stringent. It includes not only a formal examination but also the requirement that the patent demonstrates ‘outstanding substantive features and significant progress’ (Lian et al., 2022; Truong et al., 2021). This necessitates a higher degree of technical difficulty and quality. The examination process for utility model patents is relatively simpler, focusing primarily on the form and practical applicability of their technical solutions, and requiring a lower level of technical content. In terms of examination duration, the examination cycle for invention patents is longer, typically taking 2-3 years or even more, and includes both preliminary examination and substantive examination. In contrast, the examination period for utility model patents is shorter, typically lasting 6-12 months, and involves only a preliminary examination. Based on the above definition, the technical content required for green invention patents are significantly higher than that required for green utility model patents.

The measurement of SUGI is a prominent research topic in the field of green innovation and serves as the core variable of this study. However, there is currently no unified or standardized method for measuring SUGI within the academia. Many scholars use the number of green invention patent applications to measure SUGI (Lian et al., 2022; Chen et al., 2024; Zhang et al., 2023; Jiang and Bai, 2022). Yet, a few scholars employ the total number of green patent applications to measure SUGI (Liao et al., 2023). Since the total number of green patent applications reflects a company’s overall level of green innovation, it does not distinguish between specific types of patents. This may result in an overestimation of an enterprise’s SUGI. Compared to green utility model patents, green invention patents are more groundbreaking and creative in nature, higher in quality, and better at reflecting SUGI. Therefore, in line with the definition of patent law and the research of most scholars, we use the number of green invention patent applications to measure corporate SUGI. This approach has been widely recognized in academia.

Specifically, we identify green patents based on the indexed list of environmentally friendly international patent classifications published by the World Intellectual Property Organization (WIPO) in 2010. The list categorizes green technologies into seven categories: alternative energy production, transportation, energy conservation, waste management, agriculture and forestry, administrative, regulatory, or design aspects, and nuclear power generation. Following the above definition criteria, this study calculates the number of green invention patents through the following steps. First, we download information on patent applications and IPC classification numbers of listed Chinese manufacturing firms from the incoPat database. Second, based on the IPC classification numbers of green patents in the ‘International Patent Green Classification List,’ we manually search and match the IPC classification numbers of enterprises in the incoPat database with green patent information across the seven categories, thereby screening out the green patents. Third, we count and summarize the number of corporate green invention patent applications each year and used it as the measure of SUGI^[1]^.

It is worth noting that, since many applications for green invention patents are rejected due to failure to meet novelty or other requirements, the number of granted green invention patents is also a way to measure SUGI (Leyva-de la Hiz et al., 2022; Steensma et al., 2015; Hu et al., 2023). To make the measurement of SUGI more comprehensive and reliable, we use the number of granted green invention patents as a proxy indicator for SUGI. Additionally, it is recognized that enterprises may use green management techniques and methods to improve their production processes, thereby enhancing their green innovation capabilities. Such innovations may not be captured by the number of patents. Therefore, following Zhao et al. (2015), we also calculated green management innovation and used it as an alternative proxy for SUGI. In the robustness tests, we present the estimation results using the above two indicators as the explained variables.

- Thank you very much!

3) The baseline regression model (Model 1) exhibits endogeneity issues. First, omitted variable bias may contribute to endogeneity. Although the paper controls for several key variables (e.g., firm age, size, and financial leverage), there may be other unobserved factors that simultaneously influence both the Green Factory Evaluation (GFE) policy and substantive green innovation (SUGI), such as a firm’s strategic positioning or management practices. Second, endogeneity resulting from the policy selection process could also be problematic. The implementation of the GFE policy (i.e., assignment to the treatment group) may not be entirely random, as it could be influenced by unobservable firm-specific characteristics. Firms may decide to participate in the green factory certification process based on their own conditions (such as resources, technological capabilities, or prior levels of green innovation), and these characteristics are likely correlated with their green innovation outcomes. This could lead to selection bias, where better-performing firms are more likely to be designated as green factories. Third, the omission of industry and regional fixed effects may introduce additional endogeneity issues. Variations in regional subsidies for green factories and differences in policy effects across industries could distort the estimated results. To address these concerns, I recommend using instrumental variables to mitigate endogeneity and incorporating industry and regional fixed effects to improve the robustness and accuracy of the model.

- Thank you for your careful review on our manuscript. The issue you have pointed out is important. It will not only help us further enhance the reliability and robustness of our empirical findings but also provide valuable insights for refining the endogeneity analysis and improving the quality of the manuscript in future studies.
- As suggested by you, we have revised and supplemented the relevant content, incorporating the following three main components. First, to address potential endogeneity issues in the model, we selected instrumental variables for analysis and incorporated industry and region fixed effects. These additions are detailed in Section 5.3.5 Endogeneity analysis. Second, considering the influence of factors such as firms’ strategic positioning and management practices on GFE and SUGI, we selected control variables related to the board of directors and management, among others, and incorporated them into the benchmark model for regression analysis. This modification was added in Section 5.3.6 Other robustness checks. Third, to account for the potential influence of industry and regional factors on the estimation results, we incorporated industry and region fixed effects into the benchmark model. The estimation results are presented in Section 5.3.6 Other robustness checks. Specifically, the revisions are shown below (indicated by red font in the revised manuscript):

**5.3.5 Endogeneity analysis**

The enterprises announced by GFE are selected through a series of processes, such as local declaration and expert assessment. As a result, the implementation of GFE may exhibit a degree of randomization across firms. Although we control for firm and time fixed effects as well as a range of control variables, the model may still be affected by firm-time level observable or unobservable omitted variables, which could bias the regression results. To address potential endogeneity issues, we employ the two-stage least squares (2SLS) method. Following Liu et al. (2025), we select the interaction term between green space coverage and time in the province where the firm is located as an instrumental variable (IV) for GFE. First, an increase in the proportion of regional green space helps create a favorable ecological environment for the implementation of green factories, ensuring that the IV satisfies the relevance requirement. Second, regional green space is unlikely to directly affect corporate SUGI, allowing the IV to meet the exclusivity requirement. Therefore, the selection of this IV is justified.

Table 6 presents the estimation results of the IV. In Columns (1) and (2), the regression coefficients for 2SLS are 0.024 and 0.151, respectively, both of which are significantly positive at the 1% level. This indicates a strong positive correlation between GFE and the IV selected in this study, suggesting that the IV effectively explain GFE. The results of the weak IV test show that the Kleibergen-Paap rk Wald F statistic exceeds the Stock-Yogo weak IV critical value at the 10% level. Therefore, we reject the weak IV assumption and confirm the validity of this IV. After addressing the endogeneity issue, GFE still significantly promotes SUGI, which supports the core findings of this study.

**Table 6. Endogeneity analysis.**

|  | **(1)** | **(2)** |
| --- | --- | --- |
|  | **First stage** | **Second stage** |
|  | **GFE** | **SUGI** |
| **IV** | 0.024*** |  |
|  | (0.000) |  |
| **GFE** |  | 0.151*** |
|  |  | (0.058) |
| **Controls** | Yes | Yes |
| **Firm fixed** | Yes | Yes |
| **Year fixed** | Yes | Yes |
| **Industry and region fixed** | Yes | Yes |
| **N** | 15851 | 14637 |
| **Adj-R^2^** | 0.398 | 0.163 |
| **Kleibergen-Paap rk LM statistic** |  | 3.799 |
| **Cragg-Donald Wald F** |  | 6442.482 |
| **Kleibergen-Paap rk Wald F statistic** |  | 30.714  [16.38] |

Note: The values in square brackets represent the critical values at the 10% level for the Stock-Yogo weak identification test.

**5.3.6 Other robustness checks**

(4) Adding control variables. Although we control for important variables such as firm age, size, and financial leverage, there may be other unobserved factors that influence both GFE and SUGI, such as a firm’s strategic positioning or management practices. Related studies indicate that board and management characteristics significantly affect firms’ green innovation activities (Farza et al., 2022; Yousaf et al., 2024). Based on this, we incorporate additional control variables into the model, including board size, the percentage of independent directors, whether directors and supervisors have a financial background, and whether they hold dual positions. Columns (13) of Table 7 displayed the results, which support the main research conclusions.

(5) Considering industry and region fixed effects. Due to factors such as macroeconomic policies implemented in each region, inter-regional differences in GFE subsidies and the screening of pilot enterprises, as well as economic policies and cyclical changes in the manufacturing industry, there may be heterogeneous impacts on firms’ green innovation activities. To address the potential bias caused by these factors, we include both region and industry fixed effects in the benchmark model. Column (14) of Table 7 presents the estimation results, which align with the core findings of the study.

**Table 7. Other robustness tests.**

|  | **(1)** | **(2)** | **(3)** | **(4)** | **（5）** | **(6)** | **（7）** | **（8）** | **(9)** | **(10)** | **(11)** | **(12)** | **(13)** | **(14)** |
| --- | --- | --- | --- | --- | --- | --- | --- | --- | --- | --- | --- | --- | --- | --- |
|  | **SUGI2** | **SUGI3** | **SUGI4** | **SUGI5** | **SUGI6** | **Changing sample range** | | | | | **Changing model** | | **Adding control variables** | **Other fixed effects** |
| **GFE** | 0.019*** | 0.119*** | 0.116*** | 0.051** | 0.088** | 0.082*** | 0.073*** | 0.054** | 0.071** | 0.109*** | 0.082*** | 0.073*** | 0.085*** | 0.076*** |
|  | (0.005) | (0.043) | (0.051) | (0.026) | (0.035) | (0.028) | (0.026) | (0.024) | (0.031) | (0.031) | (0.028) | (0.026) | (0.027) | (0.025) |
| **Controls** | Yes | Yes | Yes | Yes | Yes | Yes | Yes | Yes | Yes | Yes | Yes | Yes | Yes | Yes |
| **Firm fixed** | Yes | Yes | Yes | Yes | Yes | Yes | Yes | Yes | Yes | Yes | Yes | Yes | Yes | Yes |
| **Year fixed** | Yes | Yes | Yes | Yes | Yes | Yes | Yes | Yes | Yes | Yes | Yes | Yes | Yes | Yes |
| **N** | 15671 | 15671 | 15671 | 15671 | 15671 | 12373 | 13617 | 11083 | 10410 | 11878 | 15671 | 15671 | 13527 | 14446 |
| **Adj-R^2^** | 0.555 | 0.609 | 0.439 | 0.601 | 0.603 | 0.645 | 0.654 | 0.686 | 0.672 | 0.658 | 0.555 | 0.439 | 0.655 | 0.654 |

- Thank you very much!

4) The authors' approach of excluding samples based solely on urban administrative structure in the robustness checks lacks methodological rigor. Instead, the authors should consider excluding regions that exhibit distinctive characteristics, such as variations in policy implementation, economic development levels, industrial structures, or green innovation capabilities, rather than arbitrarily removing samples based on administrative structure. This would ensure that the exclusions are more relevant and grounded in factors that could truly affect the results of the study.

- Thank you for your insightful review on our manuscript. The issue you pointed out is important. It not only highlights shortcomings in our empirical design of the robustness test, helping us improve the methodological rigor of this study, but also provides valuable insights into how to refine the sample selection for robustness tests in future studies.
- As suggested by you, we sequentially exclude samples that exhibit unique characteristics in terms of policy implementation, economic development level, industrial structure, and green innovation capacity, and then re-run the regression. This modification was added in Section 5.3.6 Other robustness checks. Specifically, the revisions are shown below (indicated by red font in the revised manuscript):

**5.3.6 Other robustness checks**

(2) Changing sample range. Second, considering that some regions exhibit unique characteristics in terms of policy implementation, economic development level, industrial structure, and green innovation capacity, we take the following steps to adjust the sample range to eliminate the potential interference of these factors on the research results. (i) Excluding samples from regions with fewer than 10 GFE pilot firms; (ii) To mitigate the impact of significant disparities in regional economic development on the study’s findings, we exclude samples with regional GDP per capita below the 10th percentile and above the 90th percentile. (iii) Considering that the pollution attributes of firms may influence GFE and thus bias the study’s conclusions, we exclude and re-run the regression for samples from regions where highly polluting industries account for more than 30%. (iv) To eliminate the possibility that the regional green innovation base may affect the regression results of GFE on firms’ SUGI, this study removes samples from cities with zero regional green patent applications. The results are presented in Columns (7) to (10) of Table 7. These results demonstrate that our findings are robust to factors such as regional policy implementation and economic development, further supporting the study’s conclusions. **(Note: Table 7 was shown in the response to question 3)**

- Thank you very much!

Dear Reviewer 2,

Thank you very much for giving us the opportunity to further improve the quality of our manuscript entitled “**How does China’s green factory policy affect substantive green innovation?” (PONE-D-24-52239)**. Your patient guidance has been invaluable, and we express our sincere appreciation for your valuable concerns and suggestions, which significantly contribute to improving the paper’s quality. We have read through all comments from you and tried our best to revise the entire paper as suggested. Revised portions are marked with **red words** in the paper. The main corrections and our responses to your comments are detailed as follows:

5) The research gap is not identified in a compelling way in the introduction section. IMPORTANCE, NOVELTY should be clearly stated and presented in the introduction section. Also, the authors can enrich the contributions to the existing research in the introduction section.

- Thank you for your professional review on our manuscript. The issue you pointed out is crucial. It helps us better emphasize the significance and novelty of our research and further enhances the quality of the manuscript.
- As suggested by you, we have revised and supplemented the relevant content, incorporating the following two main components. First, we have rewritten the introduction to highlight the importance of exploring the relationship between GFE and SUGI. Second, we have revised and expanded the research contributions section to emphasize the core content of our study. Specifically, the revisions are shown below (indicated by red font in the revised manuscript):

**1. Introduction**

In recent years, China’s industrialization has experienced sustained and rapid growth. While this has driven significant economic development and established a comprehensive manufacturing system, it has also resulted in increasingly severe environmental pollution issues (Wu and Guo, 2025; Yu et al., 2023; Huang et al., 2020). As a key driver in promoting green development, substantive green innovation (SUGI) provides effective support for improving environmental quality and achieving sustainable development goals. Research on SUGI is currently a key focus in academia (Han et al., 2024; Liu et al., 2024; Zhang et al., 2023; Lian et al., 2022).

However, businesses often lack the resources and incentives to promote SUGI. On the one hand, compared to traditional green innovation, SUGI involves high risk, high investment, high uncertainty, and long cycles (Shen et al., 2020), making it harder to gain support within enterprises. On the other hand, in the absence of a strict environmental policy and regulatory framework, most enterprises tend to overemphasize the ‘quantity’ of green innovations to achieve superficial environmental performance (Hu et al., 2023; Delmas and Burbano, 2011; Clarysse et al., 2009), while ignoring SUGI that genuinely contribute to the long-term sustainable development of enterprises. As a form of industrial policy aimed at environmental goals (Altenburg et al., 2017), green industrial policy can effectively overcome the limitations of traditional industrial and environmental policies in promoting green development. It serves as an effective approach to mitigating ‘market failure’ and stimulating the vitality of green innovation within enterprises (Lv and Zhang, 2024; Huang et al., 2023; Zhu and Tan, 2022; Song and Zhou, 2021; Harrison et al., 2017; Rodrik, 2014). However, existing literature has paid limited attention to how green industrial policy influences SUGI. In this context, exploring whether green industrial policy can promote corporate SUGI is highly important for accelerating the green transformation of the manufacturing industry and achieving sustainable development.

Unlike traditional environmental regulation, which focuses on end-of-pipe treatment and penalties, green industrial policy aims to guide enterprises in enhancing the research, development, and application of cleaner production technologies, as well as in developing green processes and products. One of its core goals is to promote the green transformation of enterprises. As an important component of China’s green industrial policy, the green factory evaluation (GFE), officially launched in 2016, provides a valuable opportunity to explore how green industrial policy influences corporate SUGI. In fact, some scholars have already highlighted the significant role of macro-level pilot policies (Mbanyele et al., 2024), such as the GFE, in influencing firms’ production behavior. They indicated that the GFE, as a green industrial policy, not only significantly enhances labor productivity within firms (Wei et al., 2024) but also contributes to promoting the green development of firms (Liu et al., 2025). However, these studies often focus on the economic and social impacts of the GFE or its effectiveness in promoting firms’ overall green innovation, while the systematic study of its impact on SUGI is still limited. As the core of a green manufacturing system, does the GFE promote corporate SUGI, and what are the mechanisms through which it exerts its impact? Answering this question not only helps clarify the relationship between the GFE and SUGI and offers new perspectives for exploring the theoretical study of how green industrial policy influences SUGI, but also provides more targeted practical insights for promoting the green transformation of the manufacturing industry.

Based on this, this paper seeks to systematically investigate the impact and internal logic of GFE on corporate SUGI. Specifically, based on GFE policy, we employ the time-varying difference-in-differences (TDID) model to empirically examine the link between GFE and corporate SUGI. The results show that GFE has a positive impact on promoting corporate SUGI, and the influencing mechanisms are greenwashing and industry-university-research cooperation (IURO). Further, this paper also finds that GFE significantly contributes to firms’ SUGI in industries with high energy consumption and pollution levels, as well as in firms characterized by higher levels of external information disclosure. The research conclusions provide essential evidence supporting GFE’s role in promoting the green development of micro-enterprises. Our study makes three important contributions to the existing literature:

First, this study focuses on the substantive performance of green innovation by integrating the GFE and corporate SUGI into the same research framework, offering a new perspective for exploring the drivers of SUGI. Existing studies on GFE tend to focus on exploring its impact on labor productivity or green innovation (Wei et al., 2024; Liu et al., 2025), largely ignoring its role in promoting GFE in promoting SUGI, a high-quality form of green innovation. However, this study further explores how the GFE, as a green industrial policy, promotes corporate SUGI. Our research not only contributes to the literature on the effectiveness of GFE and adds to the ongoing discussion about the efficacy of green industrial policies, but also provides new empirical evidence on how emerging manufacturing countries can effectively stimulate SUGI in the manufacturing sector.

Second, based on the characteristics of SUGI, our study conducts a comprehensive analysis of the intricate relationship between GFE and SUGI, and finds that greenwashing and IURO are the mechanisms through which GFE affects SUGI. By focusing on the information asymmetry and the resource-based theory, we demonstrate that GFE effectively addresses and alleviates the challenges of strategic green innovation and resource constraints faced by firms during the green innovation process, thereby promoting SUGI. Unlike existing studies that explore the impact mechanism of green industrial policy on SUGI from the perspectives of environmental investment and human capital investment (Xiao et al., 2024), this study further clarifies the intrinsic mechanism through which GFE promotes SUGI. These findings help unveil the ‘black box’ of GFE’s influence on SUGI through the lens of corporate behavior, thereby providing an internal logic for various market participants to explore how green industrial policies can help firms achieve SUGI goals.

Third, our study also examines how the GFE affects firms’ SUGI differently in various contexts, providing valuable empirical evidence for understanding the role of green industrial policies in guiding firms’ green innovation. Existing studies have not yet reached a consensus on how green industrial policies affect corporate green development (Wang, 2023; Tian et al., 2022; Zhu & Tan, 2022). By focusing on GFE as a green industrial policy, we find that GFE promotes SUGI in the heavy chemical industry. This provides valuable insights for heavy chemical enterprises to break path dependence and accelerate their green transformation. Additionally, we find that GFE significantly enhances SUGI when firms are subject to stronger external information disclosure. These findings provide clearer policy insights into how to effectively implement green industrial policies and facilitate the green transformation of the manufacturing industry in the current context.

- Thank you very much!

6) Comparisons with other studies have to be provided in the results discussion section. Please interpret and describe the significance of your findings in light of what was already known about the research problem being investigated and explain any new understanding or fresh insights about the problem after you've taken the findings into consideration. Please provide a comparison with other studies.

- Thank you for your insightful review on our manuscript. The issue you pointed out is highly important for improving the quality of the manuscript. A comparison between our findings and the conclusions of existing studies will help us more clearly demonstrate the significant contributions of this study and further enhance the depth and quality of our manuscript.
- As suggested by you, we have revised and expanded the discussion of the estimation results, analyzing the connections and differences between our findings and the existing studies. Specifically, the revisions are shown below (indicated by red font in the revised manuscript):

**5.2 Benchmark regression**

In fact, compared with green utility model patents, green invention patents are more groundbreaking and creative (Lian et al., 2022; Chen et al., 2024; Zhang et al., 2023; Jiang and Bai, 2022). They can effectively promote technological progress and reduce pollution emissions, serving as the real driving force to enhance the vitality of green innovation in the manufacturing industry. In the context of vigorously promoting green manufacturing, firms with GFE have strong incentives to enhance their corporate green innovation activities (Liu et al., 2025) to accelerate the realization of green transformation, particularly in SUGI focused on green invention patents.

**5.4 Mechanism test**

There is no consensus on the relationship between greenwashing and green innovation. Some studies have found that greenwashing negatively affects firms’ green innovation, particularly the quality of green innovation (Hu et al., 2023). Conversely, other studies suggest that strict environmental regulations may promote green innovation by increasing greenwashing behavior (Peng and Kong, 2024). However, we find that GFE, as a voluntary environmental regulation, can achieve long-term dynamic regulation of firms. By reducing information asymmetry between the government and firms, it mitigates strategic greenwashing behavior and incentivizes firms to engage in higher-quality and SUGI activities.

Effective integration of internal and external innovation resources, along with diverse knowledge exchanges across organizational boundaries, is essential to reinforce high levels of innovation within firms (Chesbrough, 2003). Some studies suggest that IURO enhance the role of intelligent manufacturing in promoting high-quality green innovation in firms (Dong et al., 2025). However, the aforementioned study primarily focused on the relationship between intelligent manufacturing pilot policies, IURO, and breakthrough green innovation. Our study further extends their findings. From the perspective of green industrial policy, we find that GFE can also strengthen green technology cooperation between firms and other entities, thereby fully promoting SUGI.

**5.5 Heterogeneity analysis**

Our study enriches the research on the green innovation effects of environmental policies in the heavy chemical industries. Bi et al. (2024) demonstrate that although the implementation of environmental protection laws strengthens firms’ green innovation, it does not enhance SUGI. However, our study reveals that GFE can break the path dependence of technological choices in high energy consumption and heavy pollution industries, significantly enhancing corporate SUGI.

As a critical component of the environmental governance system, environmental information disclosure serves as a key driver for promoting corporate green innovation (Zhang et al., 2024). Some scholars have highlighted the significant role of high-level disclosure in the process of green credit policies fostering corporate green innovation (Su et al., 2022). Based on this, our study finds that stronger disclosure can also synergize with GFE to jointly enhance corporate SUGI.

- Thank you very much!

7) Model construction section of your paper should clearly articulate the reasons why you chose A PARTICULAR PROCEDURE OR TECHNIQUE. The methodology should discuss the problems that were anticipated and the steps you took to prevent them from occurring, such as the heterogeneous treatment effects in running the staggered difference-in-differences regression.

- Thank you for your thorough review and valuable comments on our manuscript. Providing necessary explanations and illustrations when selecting empirical models will not only help us better explore the relationships between variables but also enhance the rigor and rationality of the study in terms of empirical model selection. These revisions will significantly improve the quality of the manuscript.
- As suggested by you, we have revised and rewritten the content, which includes the following two main parts. First, we revised the section on model construction (Section 4.3 Model construction). By analyzing the potential issues that the empirical research topic of this study may encounter, we explain the rationale for choosing the TDID model to explore the relationship between GFE and SUGI. Second, considering potential issues such as estimation bias in the use of TDID regressions, we enhance the robustness analysis by incorporating a discussion of heterogeneous treatment effects and providing the corresponding estimation results (5.3.2 Heterogeneous treatment effects). Specifically, the revisions are shown below (indicated by red font in the revised manuscript):

**4.3 Model construction**

The DID model is widely used in policy evaluation research due to its strengths in causal identification. The model can effectively control for the interference of other policies while eliminating ex ante differences between the treatment group (pilot firms) and the control group (non-pilot firms), thereby accurately estimating the net effect of policy shocks on firms’ behavior. Given that the list of GFE firms is enacted in batches, firms in the treatment group are subject to the policy at different points in time, resulting in variations in treatment timing. The TDID is suitable for studying the effects of policies implemented at different times on different individuals. It can address issues such as inconsistent treatment timing and potential estimation bias arising from traditional DID, thereby providing a more efficient estimator. Based on this, we employ the TDID model to examine the impact of GFE on firms’ SUGI. The specific model settings are as follows:

**5.3.2 Heterogeneous treatment effects**

Given that GFE policy announcements are not clustered in the same year, varying treatment points over time will subject the traditional two-way fixed effects (TWFE) to potential estimation bias (Sun and Abraham, 2021; Callaway and Sant’Anna, 2021; Goodman-Bacon, 2021). If there is heterogeneity in treatment effects, the TWFE estimator may struggle to accurately identify the true average treatment effect. For example, in our study, the total DID estimator over the sample period can be divided into the following three components: (i) New entrants to the GFE (Treatment) and those who have never been selected for the GFE (Never Treated); (ii) New entrants to the GFE (Earlier Group Treatment) and those who have not yet joined the GFE (Later Group Comparison); (iii) New entrants to the GFE (Later Group Treatment) and those who are already participating in the GFE (Earlier Group Comparison). The total DID estimator is derived as a weighted average of the basic 2$\times$2 DID estimates from the three components mentioned above. Among the three control groups, the first two serve as ‘good controls’ because they assess the impact of whether or not entering the GFE affects firms’ SUGI. However, the treatment effect in the third control is not necessarily homogeneous. If the weights assigned to the control mean estimator of the third are larger, this can influence the results of the two-way fixed effects estimator, thereby introducing bias (Goodman-Bacon, 2021; Sun and Abraham, 2021). To mitigate the bias problem in TDID estimation caused by heterogeneous treatment effects, we employ the following two methods to analyze heterogeneous treatment effects. To address the bias problem in TDID estimation caused by heterogeneous treatment effects, we employ the following two methods to analyze heterogeneous treatment effects and mitigate the limitations of the TWFE estimator.

First, considering the bias diagnosis of the estimator and Goodman-Bacon decomposition. Following Goodman-Bacon (2021), we conducted a bias diagnosis of the TWFE estimator to examine whether the third control group is overrepresented. Fig 6 presents the results of the Goodman-Bacon decomposition, indicating that the third control group constitutes a small proportion in our study. It suggests that the effect of GFE on SUGI is not driven by the third control group. In addition, we provide detailed results of the Goodman-Bacon decomposition in Table 4. It can be observed that the primary source of the overall DID estimates comes from the estimate using the never-treated sample as the control group, which accounts for up to 95% of the weight (Treatment vs. Never Treated). In contrast, the weight of the sample using those who received treatment earlier as the control group is only 0.9% (Later Treatment vs. Earlier Comparison), indicating a minimal impact on the overall estimates. These results suggest that the average treatment effects derived from the benchmark regression results in this study are not significantly biased.

Second, considering the heterogeneity-robust estimator. When the heterogeneous treatment effects are more pronounced, the dynamic effects obtained through the classical event study method may also suffer from estimation bias. To address this issue, some econometricians have employed heterogeneity-robust DID estimators to re-estimate the dynamic effects. This approach has been widely adopted in the existing literature (Braghieri et al., 2022; Liu et al., 2022; Biasi and Sarsons, 2022). Compared to the estimators proposed by De Chaisemartin and D’Haultfoeuille (2020), Callaway and Sant’Anna (2021), and others, this estimator exhibits relatively strong estimation properties and is capable of satisfying unbiasedness, validity, and consistency simultaneously. Based on this, we employ the estimation method proposed by Sun and Abraham (2021) to conduct the dynamic effects test. Fig 7 presents the estimation results, demonstrating that the parallel trend assumption remains valid after addressing the bias problem in TDID estimation caused by heterogeneous treatment effects. This confirms that the benchmark regression results in this study are robust and reliable.

**Fig 6. SUGI.**

**Table 4. Goodman-Bacon decomposition results.**

|  | **(1)** | **(2)** |
| --- | --- | --- |
|  | **SUGI** | |
| **Control Group Type** | **Weights** | **Estimated Value** |
| **Treatment vs. Never treated** | 0.954 | 0.044 |
| **Earlier Treatment vs. Later Comparison** | 0.011 | -0.084 |
| **Later Treatment vs. Earlier Comparison** | 0.009 | -0.013 |

**Fig 7. Parallel trends based on heterogeneity robust DID estimator (Sun and Abraham, 2021).**

- Thank you very much!

8) Although the authors presented the policy background and main variables empirically used in sections 4 and 5, it appears too sample and unintuitive. I would recommend the authors to detail the variables and results using more beautiful figures, which can make the work appealing for readers in standard international journals.

- Thank you for your insightful review on our manuscript. The issue you pointed out will help us better demonstrate the relevant features of GFE and SUGI during the sample period and further enhance the quality of the manuscript.

- As suggested by you, we have supplemented the relevant content, incorporating the following two main components. First, in Section 4.1.1 Green factory evaluation (GFE), we added trends in GFE over time and the distribution of GFE across manufacturing industries, along with a brief description. in Section 4.1.2 Substantive green innovation (SUGI), We added trends in SUGI over time and the distribution of SUGI across the manufacturing industry, along with a brief description. Specifically, the revisions are shown below (indicated by red font in the revised manuscript):

**4.1.1 Green factory evaluation (GFE)**

The distribution of GFE provides a better understanding of its characteristics. First, to clearly characterize the temporal evolution of GFE, we plot its time trend. Fig 1 shows the changes in the number of companies participating in GFE between 2012 and 2021. It can be observed that the number of green factories has exhibited a year-by-year increasing trend since the release of the first list of green factories. This demonstrates that the implementation of GFE has yielded positive outcomes. Its successful practice not only encourages more enterprises to actively engage in green manufacturing through the demonstration effect but also strengthens the government’s confidence and commitment to further expand the scope of the pilot program.

Second, to portray the distribution of GFE across different manufacturing industries in a more detailed manner, we mapped the industry distribution of GFE based on the Guidelines for Industry Classification of Listed Companies (revised in 2012)^[2]^. Fig 2 shows the number of firms that have been selected for GFE programs across different manufacturing sectors. It demonstrates that a higher number of companies have been approved as green factories in the following sectors: pharmaceutical manufacturing (C27), electrical machinery and equipment manufacturing (C38), manufacturing of computers, communications, and other electronic equipment (C39), chemical raw materials and chemical products manufacturing (C26), and automobile manufacturing (C36). As key industries in the transformation and upgrading of the manufacturing sector, most of these industries are characterized by high pollution and high emissions. The GFE enables these industries to ensure sustainable development while actively pursuing green transformation, further exerting a ‘point-to-point’ demonstration effect.

**Fig 1. Time trend of GFE sum.**

**Fig 2. Distribution of GFE sum in manufacturing industry.**

**4.1.2 Substantive green innovation (SUGI)**

To gain a deeper understanding of the characteristics of SUGI in manufacturing firms, we plotted the time trend and industry distribution of SUGI separately. Fig 3 shows the trend of SUGI over time. The results indicate that the overall SUGI among manufacturing firms exhibited a gradual upward trend during the sample period. However, it is worth noting that this upward trend reversed in 2018, showing a brief decline. SUGI represents a high-quality innovation behavior that requires significant R&D investment and advanced technology. Due to the shocks and uncertainties in the global economic situation, firms likely faced increased cost pressures during this period, which reduced their willingness to invest in SUGI. After 2020, SUGI resumed a gradual upward trend. With the carbon neutrality goal, enterprises are increasingly recognizing the importance of SUGI for their sustainable development and have begun to consistently strengthen their investments in cutting-edge technology and R&D. This is likely to drive an increase in SUGI activities among firms.

Fig 4 shows the distribution of SUGI across different manufacturing industries. The results indicate that the top five industries with the highest SUGI rankings are the manufacturing of computers, communications, and other electronic equipment (C39), electrical machinery and equipment manufacturing (C38), chemical raw materials and chemical products manufacturing (C26), automobile manufacturing (C36), and special equipment manufacturing (C35). Most of these industries are technology-intensive, characterized by higher R&D investment intensity and stronger technological innovation capabilities. They also perform better in the R&D and transformation of SUGI.

**Fig 3. Time trend of SUGI sum.**

**Fig 4. Distribution of SUGI sum in manufacturing industry.**

- Thank you very much!

9) Its topic appears to be within the scope of macro-level pilot policy and corporate green innovation, the authors should cite the following recent research from PLOS ONE or closed journals to connect to this ongoing discourse.

[1] https://doi.org/10.1016/j.eneco.2024.108047

[2] https://doi.org/10.1016/j.jbusres.2023.114147

[3] https://doi.org/10.1016/j.chieco.2024.102236

[4] https://doi.org/10.1016/j.techfore.2022.122007

- Thank you for your professional review on our manuscript. The issue you pointed out will help us better understand and summarize existing research findings in the field of macro-pilot policies and green innovation. The literature you provided is very important for our research, offering valuable references for studying the economic effects of green industrial policies and helping us further explore green innovation. These additions will greatly improve the completeness of our study. Thank you again for your patient review and guidance.
- As suggested by you, we have cited this valuable literature in the text to support the content of our study. The latest research from PLOS ONE and the studies mentioned above are cited in references [1],[6],[14],[19],[21],[98],[100]. Specifically, these changes are reflected in the revised manuscript and are highlighted in red font.
- Thank you very much!

10) The last section needs to be re-organized in sub-sections structure, with more insightful implications of your findings for managers and researchers (ideally in two separate sections, i.e., theoretical and practical implications). Also, the authors should clearly explain the shortcomings of this article and explore future research directions that can further expand on deification.

- Thank you for your careful review on our manuscript. The issue you pointed helps us better optimize the organization of the final section, allowing us to present the theoretical and practical implications of the research in this paper more clearly.
- As suggested by you, we have revised and rewritten the last section. In Section 6.1 Conclusion, we summarized the findings and highlighted the theoretical contributions of the study to the fields of GFE and SUGI, aiming to provide valuable theoretical insights for researchers exploring related topics in green industrial policy and green innovation. In Section 6.2 Practical implications, we propose valuable policy recommendations, aiming to provide practical insights for managers to enhance the green innovation capabilities of enterprises and formulate more targeted countermeasures. In Section 6.3 Limitation and future research, we discuss the limitations of the study and propose potential directions for future research. Specifically, the revisions are shown below (indicated by red font in the revised manuscript):

**6.1 Conclusion**

SUGI refers to a company’s long-term investment in green technology research and development, aimed at achieving breakthroughs that genuinely reduce pollutant emissions and enhance environmental benefits, thereby keeping a long-term competitive advantage for the company (Huang et al., 2022). As a core initiative to promote the green manufacturing system, GFE provides an effective approach to enhance SUGI. We treat GFE as a quasi-natural experiment and empirically examine the relationship between GFE and SUGI by using the TDID method. The main findings are summarized as follows:

First, GFE plays a significant role in promoting SUGI in manufacturing firms. Liu et al. (2025) demonstrated that GFE can enhance green innovation, providing valuable insights for our study. This study focuses on substantive technological innovation behavior within green innovation (Lian et al., 2022; Sun et al., 2024; Xiao et al., 2024). By integrating GFE and SUGI into the same research framework, we find that GFE significantly enhances firms’ SUGI. This not only enriches the discussion on the environmental performance effects of GFE but also provides new insights and directions for deepening theoretical research in the field of green innovation.

Second, after confirming the positive impact of GFE on firms’ SUGI, we further explore the mechanisms through which GFE affects SUGI. Unlike existing studies that examine the impact mechanisms of green industrial policies on SUGI in terms of environmental investment and human capital investment (Xiao et al., 2024), this study focuses on GFE and identifies the critical roles of greenwashing and IURO in promoting SUGI. Through these mechanisms, GFE addresses and mitigates issues such as strategic greenwashing and insufficient resources for green R&D faced by firms during production, significantly enhancing SUGI. These findings enrich research on the mechanisms through which GFE promotes SUGI and provide new insights and directions for exploring the channels through which macro-pilot policies influence corporate green innovation.

Third, GFE has a stronger facilitating effect on SUGI in high energy consumption and heavy pollution industries, effectively breaking the path dependence of technological choices in these sectors. David (1985) argues that path dependence in technological innovation means that prior technological innovations shape subsequent technological trajectories. This implies that in heavy chemical industries, firms’ existing stock of polluting technologies may hinder the adoption of cleaner technologies (Aghion et al., 2016), thereby obstructing green development. However, few existing studies have explored this issue from the perspective of green industrial policy. We find that GFE significantly contributes to SUGI in high energy consumption and heavy pollution industries, effectively breaking the path dependence of technological choices in these sectors. Additionally, we demonstrate that GFE significantly enhances SUGI among firms with stronger external information disclosure. This suggests that GFE can synergize with external information disclosure to promote SUGI. These findings provide new empirical evidence for researchers to analyze the role of GFE in influencing SUGI across different contexts.

**6.2** **Practical implications**

Our findings provide valuable policy suggestions for promoting green industrial policy and facilitating the green transformation of manufacturing in emerging economies.

First, the government should adopt proactive green industrial policies to strengthen corporate SUGI by promoting GFE. Our research demonstrates that GFE can significantly enhance SUGI. On the one hand, governments should develop a forward-looking, multi-level, and systematic policy framework to enhance the top-level design of green industrial policies. This will help integrate industrial policies with environmental development and provide an effective policy environment for enterprises to achieve high-quality green innovation and development. On the other hand, the government should also summarize the successful experiences of GFE, further refine and expand the scope of green factory evaluation, and fully leverage the nationwide driving effect of GFE. These measures will help provide sufficient motivational support to advance SUGI in the manufacturing industry.

Second, the government should effectively streamline the channels for implementing GFE to promote SUGI. Mechanism analysis indicates that GFE can enhance corporate SUGI by mitigating greenwashing and strengthening IURO. The government should continue to enhance the environmental regulatory system. By strengthening laws, regulations, and institutional norms, it can improve the level of environmental information disclosure, thereby reducing enterprises’ short-term strategic behaviors such as greenwashing and pseudo-green practices. In addition, the government should actively promote IURO among green factory enterprises and encourage universities, research institutions, and enterprises to establish broader green partnerships. For example, the government can encourage the establishment of industry-university-research collaboration laboratories, green technology research institutes, and green innovation platforms involving green factory enterprises, universities, and research institutions. The creation of a green cooperative ecosystem will provide pilot enterprises with access to advanced green technologies and information exchange, thereby offering an effective pathway for green factory enterprises to promote SUGI.

Third, the government should focus on fully considering the heterogeneous characteristics of enterprises and encourage heavy chemical industries to actively participate in GFE to strengthen the impact of GFE on enhancing SUGI. Our empirical results demonstrate that GFE significantly enhances corporate SUGI in heavily polluting and energy-intensive industries. Specifically, the government should formulate targeted measures to promote the green transformation of heavy chemical industries, focusing on production processes, production flows, and other aspects. This will accelerate the green transformation of the entire production chain by increasing the stock of clean technologies. For example, the government should strictly enforce GFE standards for heavy chemical industries and strengthen the supervision and management of production processes for enterprises that have been certified as green factories. This will help these industries break the path dependence on traditional technological choices, thereby enhancing SUGI.

**6.3** **Limitation and future research directions**

Our study has some limitations that suggest directions for future research. First, we use green invention patents to measure SUGI. Although we have also constructed a number of indicators to test the robustness of SUGI from different perspectives, such as the cosine similarity of green patents and the number of citations, there is not yet a broad consensus in the academic community on these measures. As the economics of innovation evolves, we will need to further explore the construction of SUGI in the future to more accurately and comprehensively reflect firms’ SUGI. Second, our study focuses on listed manufacturing companies in emerging developing countries, which may limit the generalizability of the findings. In future research, we plan to collect samples from firms in more countries and regions for comparative analysis. This will enable us to provide more comprehensive empirical evidence to identify similarities and differences in the relationship between green industrial policies and SUGI.

- Thank you very much!

1. To timely assess the impact of GFE on SUGI, following Fabrizi et al. (2018), we measure SUGI using the number of green invention patent applications with a one-period lag. Since many patents exhibit a zero and right-skewed distribution, we add one to the number of green invention patents and take the logarithm.
2. C13 = Processing of Agricultural and Sideline Products, C14 = Food Manufacturing, C15 = Manufacturing of Alcohol, Beverage and Refined Tea, C17 = Textile Industry, C18 = Textile and Garment Industry, C19 = Leather, Fur, Feather and their Products and Footwear Industry, C20 = Wood Processing and Manufacturing of Wood, Bamboo, Rattan, Palm, and Straw Products, C21 = Furniture Manufacturing, C22 = Papermaking and Paper Products Industry, C23 = Printing and Record Medium Reproduction Industry, C24 = Manufacturing of Cultural, Educational, Artistic, Sporting, and Entertainment Goods, C25 = Petroleum Processing, Coking, and Nuclear Fuel Processing Industry, C26 = Chemical Raw Materials and Chemical Products Manufacturing, C27 = Pharmaceutical Manufacturing, C28 = Chemical Fiber Manufacturing, C29 = Rubber and Plastic Products Industry, C30 = Non-Metallic Mineral Products Industry, C31 = Ferrous Metal Smelting and Rolling Processing Industry, C32 = Non-Ferrous Metal Smelting and Rolling Processing Industry, C33 = Metal Products Industry, C34 = General Equipment Manufacturing, C35 = Special Equipment Manufacturing, C36 = Automobile Manufacturing, C37 = Railway, Ship, Aerospace and Other Transportation Equipment Manufacturing, C38 = Electrical Machinery and Equipment Manufacturing, C39 = Manufacturing of Computers, Communications, and Other Electronic Equipment, C40 = Manufacturing of Instruments and Meters, C41 = Other Manufacturing, C42 = Comprehensive Utilization of Waste Resources Industry.
