# Supplementary material for: How does China’s green factory policy affect substantive green innovation?
Source: PLoS One. 2025 Jun 4;20(6):e0325438. doi: 10.1371/journal.pone.0325438 (PMC12136330; doi:10.1371/journal.pone.0325438)
Supplement: Attachment — Submitted filename: Response_to_Reviewers_auresp_2.docx [file pone.0325438.s004.docx]

Dear Reviewer 1,

Thank you very much for giving us the opportunity to further improve the quality of our manuscript entitled **“How does China’s green factory policy affect substantive green innovation?” (PONE-D-24-52239R1)**. We deeply appreciate your valuable concerns and suggestions, which enable us to further improve our paper. We have read through all comments from you and tried our best to revise the entire paper as suggested. Revised portions are marked with **red words** in the paper. The main corrections in the paper and the responses to your comments are listed as follows:

1) On the definition and representativeness of green invention patents: you have made a reasonable effort to distinguish green invention patents from utility models by referencing China’s Patent Law and the WIPO Green Classification. However, I would suggest further clarifying certain methodological choices. For instance, your reliance on the 2010 WIPO classification might introduce temporal limitations, as emerging technologies like carbon capture (prominent post-2020) could be underrepresented. Could you elaborate on the rationale for selecting the 2010 version or address its potential constraints in covering newer innovations? Additionally, while applying the WIPO framework, your analysis does not explicitly discuss whether industry-specific adjustments or sensitivity tests were conducted, particularly given sectoral clustering (e.g., energy vs. manufacturing patents). A brief discussion on the classification’s applicability across industries would strengthen your methodology.

- Thank you for your insightful and professional review on our manuscript. The issue you have pointed out is particularly important for refining our definition of green invention patents. First, your suggestions regarding patent screening criteria enable us to better justify our use of the 2010 WIPO classification for identifying green invention patents. This significantly enhances the methodological rigor and scientific validity of our approach to constructing corporate SUGI. Second, your insightful comments regarding the applicability of the WIPO classification across industries have significantly improved the manuscript’s quality while providing valuable research directions for our future studies. We have tried our best to improve the manuscript and hope that these changes will be recognized by you. We are deeply grateful for your valuable feedback.
- As suggested by you, we have thoroughly revised this section. The modifications include the following three aspects: First, we provide a detailed justification for adopting the 2010 WIPO classification. Second, we fully agree with your insight that the 2010 WIPO classification system underrepresents emerging technologies like carbon capture. We acknowledge that this is a temporal limitation of our current study. To better address this limitation, we additionally employ alternative classification criteria to identify firms’ green invention patents and incorporate these measures into our robustness checks (Section 5.3.6 Other robustness checks). Third, building on your valuable comments, we have thoroughly examined both the applicability and distinctive characteristics of the WIPO classification framework across different industrial sectors. Specifically, the revisions are shown below (indicated by red font in the revised manuscript):

**4.1.2 Substantive green innovation (SUGI)**

The rationale for selecting this classification framework is as follows: First, our study period (2012–2021) closely aligns with the temporal coverage of the 2010 WIPO classification. This version captures approximately 90% of green patents within our sample period, demonstrating strong representativeness. Second, as the first systematic green patent classification framework developed by WIPO, the 2010 IPC list encompasses approximately 200 environment-related technology categories, supported by comprehensive patent information. Its widespread adoption by global patent offices and academics from its complete IPC mapping and long-term stability. For instance, scholars such as [49-52] have applied this classification framework in their studies. Using this classification system for green patent identification helps ensure both the reliability of research findings and their international comparability.

**5.3.6 Other robustness checks**

**(1) Replacing the explained variable**

Sixth, given the limitations of the 2010 WIPO classification in identifying emerging technologies like carbon capture, this classification system may not comprehensively capture all green patents. To address this limitation, we employ the Combined Patent Classification (CPC)^[1]^ system jointly developed by the European Patent Office (EPO) and the United States Patent and Trademark Office (USPTO) to reassess firms’ green invention patent. Specific steps are as follows: First, we downloaded green invention patent applications with their CPC classification numbers for listed Chinese manufacturing firms from the incoPat patent database. Second, using CPC classification numbers for green technologies, we conducted manual searches and matches in the database to identify green invention patents under the CPC standard. Third, we aggregated annual corporate green patent applications and defined this measure as SUGI10. Column (9) of Table 7 presents the estimation results.

**Table 7. Other robustness tests.**

|  | **(1)** | **(2)** | **(3)** | **(4)** | **(5)** | **(6)** | **(7)** | **(8)** | **(9)** | **(10)** |
| --- | --- | --- | --- | --- | --- | --- | --- | --- | --- | --- |
|  | **SUGI2** | **SUGI3** | **SUGI4** | **SUGI5** | **SUGI6** | **SUGI7** | **SUGI8** | **SUGI9** | **SUGI10** | **SUGI11** |
| **GFE** | 0.019*** | 0.119*** | 0.116*** | 0.051* | 0.056* | 0.297*** | 0.016** | 0.088** | 0.144*** | 0.070*** |
|  | (0.005) | (0.043) | (0.051) | (0.029) | (0.029) | (0.065) | (0.008) | (0.035) | (0.043) | (0.027) |
| **Controls** | Yes | Yes | Yes | Yes | Yes | Yes | Yes | Yes | Yes | Yes |
| **Firm fixed** | Yes | Yes | Yes | Yes | Yes | Yes | Yes | Yes | Yes | Yes |
| **Year fixed** | Yes | Yes | Yes | Yes | Yes | Yes | Yes | Yes | Yes | Yes |
| **N** | 15671 | 15671 | 15671 | 15671 | 15671 | 13678 | 15671 | 15671 | 15671 | 15671 |
| **Adj-R^2^** | 0.555 | 0.609 | 0.439 | 0.764 | 0.741 | 0.380 | 0.478 | 0.603 | 0.637 | 0.710 |

**5.3.6 Other robustness checks**

**(1) Replacing the explained variable**

Seventh, considering that there may be differences in the applicability of green patents due to the technical characteristics of different industries, the 2010 WIPO classification framework may not precisely reflect industry clusters. For instance, patents containing keywords like ‘manufacturing’ or ‘process’ typically belong to the manufacturing patents, while those featuring ‘power generation’ or ‘scheduling’ technologies are generally classified as the energy patents. Such categorization bias may lead to the omission of green invention patents in manufacturing sectors, potentially distorting SUGI measurements. To address this limitation, we employ textual analysis methods to cross-validate and supplement the IPC classification results. Specifically, we identify industry-related keywords by screening green invention patent abstracts. When keywords such as ‘manufacturing’, ‘process’, or ‘production’ appear in an abstract, the patent is classified as a manufacturing patent; when terms including ‘power grid’, ‘energy storage’, or ‘power generation’ are detected, it is categorized as an energy patent. Ultimately, we aggregate green invention patents from manufacturing firms identified through both industry keyword screening and IPC classification, using this combined total as a proxy for SUGI, denoted as SUGI11. Column (10) of Table 7 presents the estimation results.

**Table 7. Other robustness tests.**

|  | **(1)** | **(2)** | **(3)** | **(4)** | **(5)** | **(6)** | **(7)** | **(8)** | **(9)** | **(10)** |
| --- | --- | --- | --- | --- | --- | --- | --- | --- | --- | --- |
|  | **SUGI2** | **SUGI3** | **SUGI4** | **SUGI5** | **SUGI6** | **SUGI7** | **SUGI8** | **SUGI9** | **SUGI10** | **SUGI11** |
| **GFE** | 0.019*** | 0.119*** | 0.116*** | 0.051* | 0.056* | 0.297*** | 0.016** | 0.088** | 0.144*** | 0.070*** |
|  | (0.005) | (0.043) | (0.051) | (0.029) | (0.029) | (0.065) | (0.008) | (0.035) | (0.043) | (0.027) |
| **Controls** | Yes | Yes | Yes | Yes | Yes | Yes | Yes | Yes | Yes | Yes |
| **Firm fixed** | Yes | Yes | Yes | Yes | Yes | Yes | Yes | Yes | Yes | Yes |
| **Year fixed** | Yes | Yes | Yes | Yes | Yes | Yes | Yes | Yes | Yes | Yes |
| **N** | 15671 | 15671 | 15671 | 15671 | 15671 | 13678 | 15671 | 15671 | 15671 | 15671 |
| **Adj-R^2^** | 0.555 | 0.609 | 0.439 | 0.764 | 0.741 | 0.380 | 0.478 | 0.603 | 0.637 | 0.710 |

Moreover, the manifestation of green invention patents may vary across different sectors of the manufacturing industry. For instance, in high energy consumption and heavily polluting industries, green patents tend to focus primarily on emission reduction and energy efficiency technologies, making their green attributes relatively more identifiable. In contrast, patents from low energy consumption and less polluting industries encompass a wider range of technical categories, where green technologies are often embedded in processes such as production optimization, rendering their environmental benefits comparatively more difficult to discern. This cross-industry variation in identifying green technologies may result in differential impacts of GFE on firms’ SUGI. Based on this, we categorize the manufacturing sector based on its internal characteristics, dividing the sample into HECI vs LECI and HPI vs NHPI to examine heterogeneity. Our findings reveal that GFE has a statistically significant positive effect on SUGI in HECI and HPI, whereas its impact on LECI and NHPI is insignificant. This indicates that GFE more effectively promotes corporate SUGI in industries where green technologies are more easily identifiable, compared to those where such technologies are harder to discern. These findings offer valuable insights for the WIPO classification criteria in characterizing industry differentiation by pollution attributes. (The details of this section are presented in ‘Section 5.5.1’ of the revised manuscript and indicated by red font)

Although we have employed a range of methods to re-identify green invention patents of Chinese manufacturing firms and verified the robustness of the research results. However, with the gradual deepening of research in the field of green innovation, there are more and more standards and methods for identifying green patents in academia, along with progressively richer patent data. In future research, we will endeavor to adopt higher-quality methods to comprehensively assess green invention patents across different industries and technology categories, thereby better capturing the substantive changes in green technologies.

- Thank you very much!

2) Addressing omitted variable bias: regarding robustness checks, your use of authorized invention patent counts might conflate high-impact innovations with incremental improvements. Could you consider incorporating quality indicators like patent citations or claims to better differentiate these? Furthermore, while addressing omitted variable bias, including industry×year fixed effects might more effectively control for time-varying sectoral shocks (e.g., post-2015 environmental inspections). Your current model also omits firm-level factors like CEO environmental preferences or ESG committees—exploring these in supplementary analyses could enhance robustness.

- Thank you for your insightful review on our manuscript. The issue you have pointed out provides a clearer picture of the relationship between patents and innovation, while further deepening our understanding of measurement methodologies for SUGI. Moreover, your advice on addressing omitted variable bias has been instrumental in enhancing the rigor and scientific validity of our robustness tests. We have greatly benefited from your professional review and patient guidance. Once again, we sincerely appreciate your thorough review.
- As suggested by you, we have revised and rewritten this section. The modifications include the following three aspects: First, to measure corporate SUGI more accurately, we construct quality indicators including citation counts and knowledge breadth of green invention patents, employing these as SUGI proxies in robustness tests. Second, we recognize that factors such as region, industry and time may influence firms’ green innovation activities. To mitigate potential confounding effects, we incorporate region, industry, and industry$\times$time fixed effects into the benchmark model. Third, prior studies have demonstrated that firm-level characteristics such as CEO environmental preferences and ESG committees significantly influence corporate green innovation. To address potential omitted variable bias, we incorporate these control variables into our baseline model to enhance the robustness of our findings. Specifically, the revisions are shown below (indicated by red font in the revised manuscript):

**4.1.2 Substantive green innovation (SUGI)**

Notably, some studies suggest that compared to the quantity of green patent applications or grants, the citation status of corporate green patents can reflect the innovation value and quality of green technologies [53]. To ensure a more comprehensive and reliable measurement of SUGI, we applied quality-based indicators such as citation counts and knowledge breadth of green invention patents as alternative variables for SUGI.

**5.3.6 Other robustness checks**

**(1) Replacing the explained variable**

Fourth, since patent citations and knowledge breadth can reflect the quality of firms’ green innovation [53], following [68,72-75], we measured corporate SUGI through four indicators: (i) The natural logarithm of the cumulative number of citations plus one for green invention patents applied by firms, namely SUGI5. (ii) The natural logarithm of the cumulative citations number of green invention patents applied by firms with self-citation exclusion plus one, namely SUGI6. (iii) The ratio of the number of citations of green invention patents applied by enterprises and that in the industries where the enterprises belong, we define it as SUGI7. (iv) We calculated the knowledge breadth of green invention patents and regard it as SUGI8^[2]^. Columns (4) to (7) of Table 7 present the estimation results.

**Table 7. Other robustness tests.**

|  | **(1)** | **(2)** | **(3)** | **(4)** | **(5)** | **(6)** | **(7)** | **(8)** | **(9)** | **(10)** |
| --- | --- | --- | --- | --- | --- | --- | --- | --- | --- | --- |
|  | **SUGI2** | **SUGI3** | **SUGI4** | **SUGI5** | **SUGI6** | **SUGI7** | **SUGI8** | **SUGI9** | **SUGI10** | **SUGI11** |
| **GFE** | 0.019*** | 0.119*** | 0.116*** | 0.051* | 0.056* | 0.297*** | 0.016** | 0.088** | 0.144*** | 0.070*** |
|  | (0.005) | (0.043) | (0.051) | (0.029) | (0.029) | (0.065) | (0.008) | (0.035) | (0.043) | (0.027) |
| **Controls** | Yes | Yes | Yes | Yes | Yes | Yes | Yes | Yes | Yes | Yes |
| **Firm fixed** | Yes | Yes | Yes | Yes | Yes | Yes | Yes | Yes | Yes | Yes |
| **Year fixed** | Yes | Yes | Yes | Yes | Yes | Yes | Yes | Yes | Yes | Yes |
| **N** | 15671 | 15671 | 15671 | 15671 | 15671 | 13678 | 15671 | 15671 | 15671 | 15671 |
| **Adj-R^2^** | 0.555 | 0.609 | 0.439 | 0.764 | 0.741 | 0.380 | 0.478 | 0.603 | 0.637 | 0.710 |

**(5) Considering other fixed effects**

To address the potential biases caused by these factors, we incorporate region, industry and industry$\times$year fixed effects into the baseline model. Column (9) of Table 8 presents the estimation results, which align with the study’s core findings.

**Table 8. Other robustness tests.**

|  | **(1)** | **(2)** | **(3)** | **(4)** | **(5)** | **(6)** | **(7)** | **(8)** | **(9)** |
| --- | --- | --- | --- | --- | --- | --- | --- | --- | --- |
|  | **Changing sample range** | | | | | **Changing model** | | **Adding control variables** | **Other fixed effects** |
| **GFE** | 0.082*** | 0.073*** | 0.105*** | 0.071** | 0.104*** | 0.082*** | 0.073*** | 0.085*** | 0.074*** |
|  | (0.028) | (0.026) | (0.032) | (0.031) | (0.031) | (0.028) | (0.026) | (0.029) | (0.024) |
| **Controls** | Yes | Yes | Yes | Yes | Yes | Yes | Yes | Yes | Yes |
| **Firm fixed** | Yes | Yes | Yes | Yes | Yes | Yes | Yes | Yes | Yes |
| **Year fixed** | Yes | Yes | Yes | Yes | Yes | Yes | Yes | Yes | Yes |
| **N** | 12373 | 13617 | 11809 | 10410 | 12059 | 15671 | 15671 | 12374 | 14446 |
| **Adj-R^2^** | 0.645 | 0.654 | 0.657 | 0.672 | 0.657 | 0.555 | 0.439 | 0.660 | 0.657 |

**(4) Adding control variables**

Furthermore, considering the CEO’s environmental background, executives’ green perceptions, and the presence of ESG committees may influence corporate governance and strategy, these factors ultimately affect green innovation [78]. Based on this, we incorporate additional control variables into the model, including board size, the percentage of independent directors, whether directors and supervisors have a financial background, whether they hold dual positions, CEOs’ environmental preferences^[3]^, executives’ green perceptions^[4]^ and ESG committee^[5]^. Columns (8) of Table 8 displayed the results, which support the main research conclusions.

**Table 8. Other robustness tests.**

|  | **(1)** | **(2)** | **(3)** | **(4)** | **(5)** | **(6)** | **(7)** | **(8)** | **(9)** |
| --- | --- | --- | --- | --- | --- | --- | --- | --- | --- |
|  | **Changing sample range** | | | | | **Changing model** | | **Adding control variables** | **Other fixed effects** |
| **GFE** | 0.082*** | 0.073*** | 0.105*** | 0.071** | 0.104*** | 0.082*** | 0.073*** | 0.085*** | 0.074*** |
|  | (0.028) | (0.026) | (0.032) | (0.031) | (0.031) | (0.028) | (0.026) | (0.029) | (0.024) |
| **Controls** | Yes | Yes | Yes | Yes | Yes | Yes | Yes | Yes | Yes |
| **Firm fixed** | Yes | Yes | Yes | Yes | Yes | Yes | Yes | Yes | Yes |
| **Year fixed** | Yes | Yes | Yes | Yes | Yes | Yes | Yes | Yes | Yes |
| **N** | 12373 | 13617 | 11809 | 10410 | 12059 | 15671 | 15671 | 12374 | 14446 |
| **Adj-R^2^** | 0.645 | 0.654 | 0.657 | 0.672 | 0.657 | 0.555 | 0.439 | 0.660 | 0.657 |

- Thank you very much!

3) Refining sample exclusion criteria: your exclusion criteria based on cross-sectional thresholds (e.g., single-year GDP percentiles) might overlook persistent confounders. Would expanding these to a dynamic framework (e.g., excluding cities with no green patents for three consecutive years) better address such concerns? Addressing these points would further clarify your study’s rigor and generalizability.

- Thank you for your insightful review on our manuscript. The issue you have pointed out is very important. We fully endorse your suggestion to expand the sample exclusion test to a dynamic framework for robustness analysis. These adjustments not only allow for a more precise assessment of GFE policy effects and enhance the study’s rigor and reliability, but also provide valuable research directions for our future empirical analyses. We sincerely appreciate your expert review and guidance throughout this process.
- As suggested by you, we have revised and rewritten the “(2) Changing sample range” section. The modifications include the following two aspects: First, we excluded observations with regional GDP per capita growth rates below the 10th percentile and above the 90th percentile. Second, we excluded cities with zero green patent applications for three consecutive years and re-estimated the regression model. Specifically, the revisions are shown below (indicated by red font in the revised manuscript):

**5.3.6 Other robustness checks**

**(2) Changing sample range**

(ii) To mitigate the influence of regional economic development dynamics, we excluded observations with regional GDP per capita growth rates below the 10th percentile and above the 90th percentile. The results are presented in Column (3) of Table 8.

(iv) Given that the effects of green innovation require time to materialize and some cities exhibit persistent non-innovation, we excluded cities with zero green patent applications for three consecutive years and re-estimated the regression model. The results are presented in Column (5) of Table 8. These results demonstrate that our findings are robust to factors such as regional policy implementation and economic development, further supporting the study’s conclusions.

**Table 8. Other robustness tests.**

|  | **(1)** | **(2)** | **(3)** | **(4)** | **(5)** | **(6)** | **(7)** | **(8)** | **(9)** |
| --- | --- | --- | --- | --- | --- | --- | --- | --- | --- |
|  | **Changing sample range** | | | | | **Changing model** | | **Adding control variables** | **Other fixed effects** |
| **GFE** | 0.082*** | 0.073*** | 0.105*** | 0.071** | 0.104*** | 0.082*** | 0.073*** | 0.085*** | 0.074*** |
|  | (0.028) | (0.026) | (0.032) | (0.031) | (0.031) | (0.028) | (0.026) | (0.029) | (0.024) |
| **Controls** | Yes | Yes | Yes | Yes | Yes | Yes | Yes | Yes | Yes |
| **Firm fixed** | Yes | Yes | Yes | Yes | Yes | Yes | Yes | Yes | Yes |
| **Year fixed** | Yes | Yes | Yes | Yes | Yes | Yes | Yes | Yes | Yes |
| **N** | 12373 | 13617 | 11809 | 10410 | 12059 | 15671 | 15671 | 12374 | 14446 |
| **Adj-R^2^** | 0.645 | 0.654 | 0.657 | 0.672 | 0.657 | 0.555 | 0.439 | 0.660 | 0.657 |

- Thank you very much!

Dear Reviewer 2,

Thank you very much for your careful and professional review on **“How does China’s green factory policy affect substantive green innovation?” (PONE-D-24-52239R1)**. Your comments and suggestions were invaluable and they greatly improved the quality of the paper. Your endorsement helps us tremendously! Once again, we would like to express our gratitude to you for your patience and guidance. Have a nice day!

1. The CPC generally adheres to the classification principles of the IPC while introducing nine divisions (adding division Y to the existing A-H divisions), primarily designed to accommodate emerging technologies and interdisciplinary technologies. Specifically, Y02 is exclusively designated for climate change mitigation and adaptation technologies, serving as a reference standard for identifying green patents. The Y02 classification comprises: Y02A (climate change adaptation technologies), Y02B (building-related energy efficiency technologies), Y02C (carbon capture and sequestration), among others. For detailed information, please refer to: <https://www.cooperativepatentclassification.org/cpcSchemeAndDefinitions/table>. The CPC classification enables more comprehensive identification of emerging green technologies, thereby compensating for the IPC system’s limitations in classifying green patents.
2. The calculation formula is:

$${SUGI8}_{it}=1-\sum a^{2}$$

   ${SUGI8}_{it}$ represents the knowledge breadth of firm $i$’s green invention patents in year $t$. $a$ represents the proportion of each main group in green invention patent classification numbers. A higher value of ${SUGI8}_{it}$ indicates broader knowledge integration in the green invention patent and consequently higher patent quality. After calculating the knowledge breadth of each green invention patent, we aggregated the measures to the firm level by computing the average values, thereby obtaining the final knowledge breadth of green invention patents.
3. Following [79-80], we constructed the CEOs’ environmental preferences. A CEO’s environmental preference is assigned a value of 1 if the CEO possesses green work or educational experience, and 0 otherwise. Specifically, a CEO is considered to have green work experience if their professional background or roles involve environmental protection, energy conservation, or corporate pollution prevention and control. A CEO is considered to have green educational experience if they majored in pulp and paper science, environmental studies, environmental engineering, environmental science, or any other environment-related discipline. The relevant data are sourced from the executive profile module of the CSMAR database.
4. Following [81]’s methodology for measuring executive cognition, we conducted textual analysis of Chinese listed companies’ annual reports. We selected relevant keywords for frequency counting and ultimately constructed a measure of corporate executives’ green perceptions. The keywords include: energy conservation and emission reduction, environmental strategy, environmental philosophy, environmental management organization, environmental education, environmental training, environmental technology development, environmental audit, energy saving and environmental protection, environmental policy, environmental department, environmental inspection, low-carbon environmental protection, environmental work, environmental governance, environmental protection and governance, environmental facilities, environmental laws and regulations, and pollution control.
5. The ESG committee serves as a key mechanism for the board of directors to address corporate environmental and social matters. Following [82], we calculated a dummy variable for whether the company has an ESG committee. Since the vast majority of companies have not yet established dedicated ESG or CSR committees—instead assigning these responsibilities to committees related to sustainability, environmental protection, or stakeholder rights. Based on this, we expanded our sample selection criteria. We define ESG committee as 1 if a company has any committee whose title or responsibilities include terms such as ESG, CSR, social responsibility, sustainability, green, environmental protection, consumer rights protection or information disclosure, and 0 otherwise.
